# Genome sequence of *Emydomyces testavorans* type strain 16-2883 (ATCC TSD-145), an onygenalean fungus associated with freshwater aquatic turtle shell lesions

**DOI:** 10.1128/MRA.00222-23

**Published:** 2023-08-08

**Authors:** Lois L. Hoyer, Elizabeth K. Hogan, Laura Adamovicz, Matthew C. Allender, Karen A. Terio, Alvaro G. Hernandez

**Affiliations:** 1 Department of Pathobiology, College of Veterinary Medicine, University of Illinois Urbana-Champaign, Urbana, Illinois, USA; 2 Roy J. Carver Biotechnology Center, University of Illinois Urbana-Champaign, Urbana, Illinois, USA; 3 Wildlife Epidemiology Laboratory, Department of Veterinary Clinical Medicine, College of Veterinary Medicine, University of Illinois Urbana-Champaign, Urbana, Illinois, USA; 4 Veterinary Diagnostic Laboratory, College of Veterinary Medicine, University of Illinois Urbana-Champaign, Urbana, Illinois, USA; 5 Chicago Zoological Society, Brookfield Zoo, Brookfield, Illinois, USA; 6 Zoological Pathology Program, Department of Veterinary Clinical Medicine, College of Veterinary Medicine, University of Illinois Urbana-Champaign, Brookfield, Illinois, USA; University of Maryland School of Medicine, Baltimore, Maryland, USA

**Keywords:** fungus, turtle, shell disease, Onygenales, genome sequence, annotation, keratin

## Abstract

*Emydomyces testavorans* is an onygenalean keratinophilic fungus associated with shell and skin lesions in freshwater aquatic turtles. The genome sequence presented here includes five contigs (ranging in size from 2.8 to 9.8 Mb; 31.8 Mb total; 40% GC) and a 92.2-kb mitochondrial genome. The nuclear genome predicted 7,550 genes.

## ANNOUNCEMENT

The keratinophilic fungus *Emydomyces testavorans* (order Onygenales) is associated with disease in free-ranging and captive freshwater aquatic turtles (1). *E. testavorans* isolate 16-2883 was collected from a western pond turtle (*Actinemys marmorata*) in the state of Washington, USA, in 2016. The culture was deposited in the American Type Culture Collection under accession number TSD-145. Taxonomic identification and the associated genetic sequences, methods for *in vitro* cultivation, and MIC values for antifungal drugs were published previously (2).

*E. testavorans* strain 16-2883 was cultured on turtle keratin medium (TKM; per liter: 2% dextrose, 1% peptone, 1% ground turtle shell scutes, with 2% Bacto agar added for plates [2]). The *E. testavorans* stock culture was maintained at −80°C in TKM with 38% glycerol. The fungus was streaked to a TKM agar plate and incubated at room temperature (20°C–22°C) until colonies developed (approximately 14 days).

Twenty milliliters of TKM (without antibacterial or antifungal agents) were placed in a 50-mL sterile, glass Erlenmeyer flask with a loosened screw top cap. A fungal colony was scraped from the TKM stock plate and placed into the liquid medium. The flask was incubated at room temperature without shaking for 14 days. *E. testavorans* cells were collected by centrifugation at 3,000 × *g*. Multiple 50-mL flask cultures were processed to collect enough genomic DNA for sequencing efforts.

Genomic DNA was extracted using a zymolyase-based protocol (3). DNA was treated with RNase, then with proteinase K (both from Gold Biotechnology), and then purified using the MagAttract HMW DNA kit (Qiagen). High-molecular-weight genomic DNA (>50 kb) was verified by visualization on an ethidium-bromide-stained agarose gel. Genomic DNA was sheared to an average fragment length of approximately 10 kb using Megaruptor 3 (Diagenode). The sheared DNA was size selected for fragments 3 to 50 kb on a BluePippin system with a 0.75% gel cassette and DNA marker S1 (Sage Science). The sequencing library was made using the SMRTbell Express Template Prep Kit 2.0 (Pacific Biosciences). The library was sequenced on a SMRTcell 8M on a PacBio Sequel IIe system using a Sequel II Binding Kit 3.2, the circular consensus sequencing (CCS) mode, and a 30-hour movie time. CCS and demultiplexing analysis were done using SMRT Link v11.0 with the following parameters: ccs –min-passes 3 –min-rq 0.99; lima –hifi-preset SYMMETRIC –split-bam-named –peek-guess. The data set included 929,480 reads with a read *N*_50_ of 11,112 bases.

Sequence reads were filtered to retain 75% of those with a minimum length of 15,000 nt (Filtlong v0.2.1 [4]. The filtered data set targeted 50× coverage of the predicted 32 Mb genome size. The genome assembly was produced with hifiasm v0.16.1 (5) using default parameters. The resulting primary contigs were deposited into the NCBI database and were the basis for subsequent analysis and annotation.

The nuclear assembly included five contigs (9.8, 9.6, 5.1, 4.5, and 2.8 Mb) with a total length of 31,798,767 bp and a GC content of 40.2%. Telomeric repeats (TTAGGG) were present on the ends of the contigs except for the 3′ end of the 9.6 and 5.1 Mb fragments and the 5′ end of the 4.5 Mb fragment. Presently, the *E. testavorans* karyotype is unknown. The hifiasm program produced a circular predicted mitochondrial genome sequence of 92.2 kb.

BUSCO v5.3.2 (6) was use to assess the nuclear genome assembly. The percent complete and single-copy BUSCOs were 98.4% (fungi_odb10), 97.1% (ascomycota_odb10), 94.7% (eurotiomycetes_odb10), and 95.7% (onygenales_odb10). Gene prediction for the nuclear genome used funannotate v.1.8.13 (7). The assembly was prepared using funannotate clean, funannotate sort, and funannotate mask, each with default parameters. Gene prediction used funannotate predict without GeneMark. No other evidence was provided to the script. Annotation was accomplished using funannotate annotate. Output files from eggNOG-mapper web v.2.1.9 [(8, 9); eggNOG5 database] and InterProScan v5.56-89.0 (-dp -goterms -pa [10]) were passed to the script. The nuclear genome predicted 7,550 genes.

## Data Availability

This whole-genome sequencing and assembly project was deposited in GenBank (PRJNA931726). Sequence reads were deposited in the Sequence Read Archive (SRR23354024). The project was assigned the locus prefix tag PRK78 (SAMN33096649).
